# Cre-mediated recombination efficiency and transgene expression patterns of three retinal bipolar cell-expressing Cre transgenic mouse lines

**Published:** 2013-06-12

**Authors:** Qi Lu, Elena Ivanova, Tushar H. Ganjawala, Zhuo-Hua Pan

**Affiliations:** Department of Ophthalmology and Anatomy & Cell Biology, Wayne State University School of Medicine, Detroit, MI

## Abstract

**Purpose:**

Retinal bipolar cells, comprising multiple types, play an essential role in segregating visual information into multiple parallel pathways in the retina. The ability to manipulate gene expression in specific bipolar cell type(s) in the retina is important for understanding the molecular basis of their normal physiological functions and diseases/disorders. The Cre/LoxP recombination system has become an important tool for allowing gene manipulation in vivo*,* especially with the increasing availability of cell- and tissue-specific Cre transgenic mouse lines. Detailed in vivo examination of the Cre/LoxP recombination efficiency and the transgene expression patterns for cell- and tissue-specific Cre transgenic mouse lines is essential for evaluating their utility. In this study, we investigated the Cre-mediated recombination efficiency and transgene expression patterns of retinal bipolar cell-expressing Cre transgenic lines by crossing with a Cre reporter mouse line and through Cre-dependent recombinant adeno-associated virus (rAAV) vector-mediated transgene delivery.

**Methods:**

Three retinal bipolar cell-expressing Cre-transgenic mouse lines, 5-HTR2a-cre, Pcp2-cre, and Chx10-cre, were crossed with a strong Cre reporter mouse line that expresses a red fluorescent protein variant, tdTomato. rAAV2 vectors carrying a double-floxed inverted open-reading frame sequence encoding channelrhodopsin-2-mCherry (ChR2-mCherry) driven by a ubiquitous neuronal EF1α or a ubiquitous CMV promoter with a rAAV2 capsid mutation (Y444F) were injected into the intravitreal space of the eyes. Immunohistochemistry using retinal bipolar cell type–specific markers was performed to examine Cre-mediated recombination efficiency and the transgene expression patterns in bipolar cells in retinal whole mounts and vertical sections.

**Results:**

For the 5-HTR2a-cre and Pcp2-cre mouse lines, the expression pattern of the Cre-mediated recombination by crossing the reporter line largely resembled the expression pattern of Cre. The bipolar cells showing Cre-mediated recombination in the 5-HTR2a-cre line and the Pcp2-cre line were predominantly type 4 cone bipolar cells and rod bipolar cells, respectively. For the Chx10-cre mouse line, the expression pattern of the Cre-mediated recombination by crossing the reporter line was different from that of Cre. The Cre-mediated transgene expression in retinal bipolar cells in the Chx10-cre line was not observed by crossing with the reporter mouse line but through Cre-dependent rAAV vector delivery. A rAAV2 vector with the combination of a CMV promoter and the Y444F capsid mutation achieved Cre-dependent transgene expression in retinal bipolar cells.

**Conclusions:**

The retinal bipolar cell-expressing Cre-transgenic lines and the Cre-dependent rAAV vector reported in this study could be valuable tools for gene targeting and manipulation in retinal bipolar cells in mice.

## Introduction

Retinal bipolar cells, second-order neurons in the retina, transmit visual information from photoreceptors to third-order retinal neurons. Bipolar cells, comprising multiple types, play an essential role in segregating visual information into multiple parallel pathways in the retina [1]. Bipolar cells are subdivided into ON and OFF types, based on the cells’ light-response polarity, and into rod and cone bipolar cells, based on the cells’ synaptic inputs. In mammals, a single type of rod bipolar cell [2,3] and at least ten types of cone bipolar cells have been characterized based on the terminal stratification in the inner plexiform layer (IPL) and cell-type-specific molecular markers [4-11]. Bipolar cells of different types exhibit diverse physiological properties [12-15]; however, less is known about the molecular basis of this diversity.

The ability to manipulate gene expression in specific bipolar cell type(s) in the retina is important for understanding the molecular mechanisms of the cells’ normal physiologic properties and diseases/disorders related to bipolar cell dysfunction, as well as for developing animal models for gene therapy targeted to bipolar cells. The Cre/LoxP recombination system has become a powerful tool for allowing gene manipulation in vivo [16,17], especially with the increasing availability of cell- and tissue-specific Cre transgenic mouse lines [18,19]. A widely used conditional gene-targeting approach is to cross cell- and tissue-specific Cre transgenic mouse lines with Cre-dependent reporter or conditional mouse lines. Cre transgenic lines, especially those produced by conventional methods (via pronuclear injection), are subject to the local chromatin environment (i.e., position effect), which could lead to transgene silencing or variable ectopic expression [20-23]. Detailed in vivo examination of the expression pattern and recombination efficiency of Cre-mediated reporter gene expression in targeted tissues is essential for evaluating their utility. Cre-dependent virus-mediated gene delivery is another powerful approach that can be used to target a transgene to Cre-expressing cells in transgenic mouse lines [24-26]. Recombinant adeno-associated virus (rAAV) vectors have been particularly widely used in retinal gene transfer [27]. However, retinal bipolar cells, due to their anatomic location in the middle of the retina, are the most inaccessible cell types in the retina for virus transduction. The ability of the Cre-dependent rAAV vector-mediated transgene delivery to retinal bipolar cells in Cre transgenic mouse lines has not been examined.

Thus far, only a small number of retinal bipolar cell-expressing Cre transgenic mouse lines have been reported [28-33]. Most were driven by the Purkinje cell protein 2 (Pcp2) promoter, a gene known to target Purkinje cells in the cerebellum as well as in retinal rod bipolar cells [34]. Few Cre transgenic lines have been reported to target retinal cone bipolar cells. One is Chx10-cre, which was reported to target the Cre recombinase in multiple retinal bipolar cell types [29]. In this study, we examined the Cre-mediated recombination expression profiles of three retinal bipolar cell-expressing Cre-transgenic lines, 5-HTR2a-cre, Pcp2-cre, and Chx10-cre, by crossing these lines to a strong Cre reporter mouse line. We characterized the Cre-mediated expression patterns in multiple bipolar cell types in the 5-HTR2a-cre and Pcp2-cre mouse lines. We also examined the ability and transduction efficiency of Cre-dependent rAAV-mediated transgene delivery to bipolar cells of the bipolar cell-expressing transgenic lines. We found that a Cre-dependent rAAV2/2 vector using a cytomegalovirus (CMV) promoter and containing a capsid mutation of Y444F can achieve Cre-mediated transgene expression in retinal bipolar cells.

## Methods

### Animals

All animal handling procedures were approved by the Institutional Animal Care and Use Committee at Wayne State University and were in accordance with the NIH Guide for the Care and Use of Laboratory Animals. Three Cre transgenic lines were used in this study: (1) Tg(Htr2a-cre)KM207Gsat/Mmcd (referred to as 5-HTR2a-cre), produced by the expression of Cre under the control of the BAC 5-hydroxytryptamine (serotonin) receptor 2A promoter [18,35]; (2) Tg(Pcp2-cre)1Amc/J (referred to as Pcp2-cre), generated by driving Cre recombinase and GFP under the control of a modified mouse Purkinje cell protein 2 (Pcp2) promoter enhancer [36]; and (3) Tg(Chx10-EGFP/cre-ALPP)2Clc/J (referred to as Chx10-cre), produced by the expression of a GFP/Cre fusion transgene under the control of a BAC clone containing the mouse transcription factor Chx10 (*C. elegans* ceh-10 homeodomain-containing homolog) promoter [29]. The 5-HTR2a-cre line was chosen because a previous study showed that a 5-HTR2a-GFP line produced by the expression of GFP under the control of the same BAC 5-HTR2a promoter predominantly expressed GFP in type 4 cone bipolar cells [37]. The transgenes of the Pcp2-cre and Chx10 mouse lines contain GFP, but the GFP fluorescence signal is relatively weak [32]. These mice were crossed with a strong Cre reporter line, B6.Cg-Gt(ROSA)26Sortm9(CAG-tdTomato)Hze/J (referred to as the tdTomato reporter line), which harbors a combination of the CMV early enhancer element and chicken beta-actin (CAG) promoter-driven red fluorescent protein (RFP) variant, tdTomato, at the Gt(ROSA)26Sor locus with a loxP-flanked STOP sequence [38]. When bred to mice with a Cre recombinase gene under the control of a promoter of interest, the STOP sequence is deleted in Cre-expressing cells, and tdTomato is expressed. The 5-HTR2a-cre mice were purchased from Mutant Mouse Regional Resource Center (MMRRC; University of California, Davis, CA); all other mice were purchased from Jackson Laboratory (Bar Harbor, ME).

### Recombinant adeno-associated virus construct and virus injection

The construct that conferred the rAAV2-mediated expression of a double-floxed inverted open-reading frame (DIO) encoding channelrhodopsin-2-mCherry (ChR2-mCherry) with a ubiquitous neuronal promoter elongation factor I alpha (EF1α), rAAV2-EF1α-DIO-ChR2-mCherry, was kindly provided by Karl Deiserroth at Stanford University (Stanford, CA). The construct of rAAV2-CMV-DIO-ChR2-mCherry was made by replacing EF1α with the ubiquitous viral promoter, CMV. Virus vectors of rAAV2-EF1α-DIO-ChR2-mCherry were produced in normal rAAV2, and virus vectors of rAAV2-CMV-DIO-ChR2-mCherry were made in a Y444F capsid mutation at the virus core facility at University of Pennsylvania (Philadelphia, PA). Virus vectors were injected intravitreally into the eyes of Cre-transgenic mouse lines, as previously described [32]. Briefly, 1- to 2-month-old mice were anesthetized by intraperitoneal injection of a mixture of 120 mg/kg ketamine and 15 mg/kg xylazine. Under a dissecting microscope, a small perforation was made in the temporal sclera region with a needle. A total of 1.5 μl of viral vector suspension in saline at a concentration of 1.3×10^12^ GC/ml and 5.6×10^12^ GC/ml for rAAV2(Y444F)-CMV-DIO-ChR2-mCherry and rAAV2-EF1α-DIO-ChR2-mCherry, respectively, was injected into the intravitreal space through the perforation with a Hamilton syringe. ChR2-mCherry expression was examined 1 month after the injection.

### Immunohistochemical staining

Mice were deeply anesthetized with CO_2_ and decapitated. The retinas were fixed in eyecups with 4% paraformaldehyde in 0.1 M phosphate buffer (PB; pH 7.4) for 20 min. For retinal whole mounts, the fixed retina was dissected free in PB solution, flat mounted on slides, and coverslipped. For retinal vertical sections, the retinas were cryoprotected in a sucrose gradient (10%, 20%, and 30% w/v in PB), and cryostat sections were cut at 14 μm.

The expression of tdTomato fluorescence in the retina was examined in retinal whole mounts and/or vertical sections. For retinal vertical section imaging, tdTomato fluorescence was sufficient to visualize the tdTomato signal. Therefore, the tdTomato fluorescence was not enhanced with an antibody. For retinal whole-mount imaging, to stabilize the fluorescence signal and reduce bleaching, the tdTomato fluorescence was enhanced using an anti-mCherry antibody, which recognizes tdTomato, followed by a secondary antibody conjugated to Alexa 555 or Alexa 594 (Molecular Probes, Carlsbad, CA).

For immunostaining, retinal whole mounts or vertical sections were blocked for 1 h in a solution containing 5% Chemiblocker (membrane-blocking agent; Chemicon, Brica, MA), 0.5% Triton X-100, and 0.05% sodium azide, Sigma). The primary antibodies were diluted in the same solution and applied overnight, followed by incubation (1 h) in the secondary antibodies, which were conjugated to Alexa 594 (1:600, red fluorescence), Alexa 555 (1:600, red fluorescence), Alexa 488 (1:600, green fluorescence, Molecular Probes), or AMCA (1:200; 711-155-152, blue fluorescence, Jackson Laboratory, Bar Harbor, ME). The following antibodies were used in this study: rabbit anti-mCherry (1:500; 632,496, Clontech, Mountain View, CA); mouse anticalsenilin (1:2,000; kindly provided by W. Wasco, Harvard Medical School, Boston, MA); rabbit anti-PKC (1:20,000; catalog number 2056, Cell Signal, Danvers, MA); mouse anti-PKC (1:10000; catalog number sc8393, Santa Cruz, CA); mouse antisynaptotagmin II (Syt2; 1:600; Zebrafish International Resource Center, Eugene, OR); rabbit anti-HCN4 (1:500; Alomone Labs, Jerusalem, Israel); and mouse antiprotein kinase A (PKA) RIIβ (1:80,000; BD Biosciences, San Jose, CA).

All steps were performed at room temperature (RT). All images were made using a Zeiss Axioplan 2 microscope (Carl Zeiss, Oberkochen, Germany) with the Apotome oscillating grating to reduce out-of-focus stray light. Z-stack images (0.5 μm step size, 20 to 30 sections) were captured and displayed as maximum intensity projections. The full image size was 1388×1040 pixels. The excitation light source was mercury short-arc HXP 120. The following filter sets (excitation, dichroic mirror, and emission) were used: Alexa 555 or 594 (band pass filter [BP] 546/12, dichroic beam splitter mirror [FT] 580, long pass filter [LP] 590); Alexa 488 (BP 470/40, FT 510, and BP 540/50); AMCA (labeling, BP 365/12, FT 395, LP 397). The brightness and contrast of the final images were adjusted using Adobe Photoshop CS4 to enhance visibility.

## Results

Three retinal bipolar cell-expressing Cre transgenic lines, 5-HTR2a-cre, Pcp2-cre, and Chx10-cre, were crossed with a strong Cre-dependent reporter line that expresses an RFP variant, tdTomato, to examine the Cre-medicated recombination pattern in the retina and especially in bipolar cells. The expression of tdTomato fluorescence was examined in retinal whole mounts with antibody enhancement while in retinal vertical sections without antibody enhancement (see Methods).

### 5-HTR2a-cre mouse line

**Figure 1:**
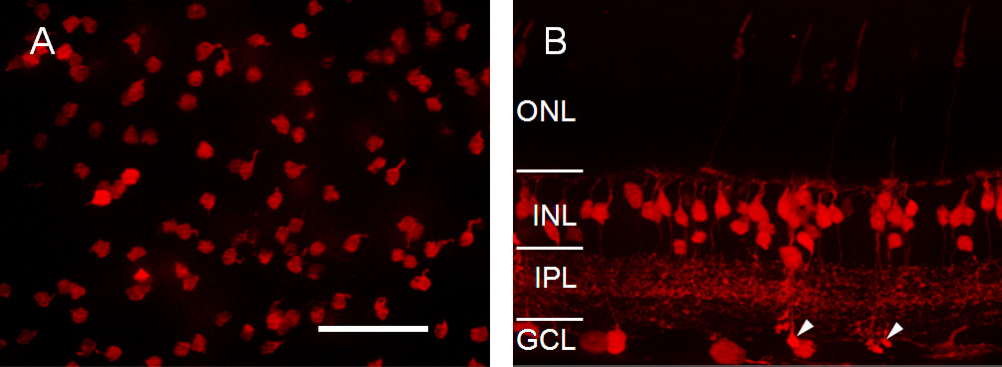
tdTomato-expressing bipolar cells in the 5-HTR2a-cre transgenic mouse line. **A**: tdTomato-expressing cells viewed in a retinal whole mount with the focal plane at the distal portion of the INL. **B**: tdTomato-expressing cells viewed in a retinal vertical section. The arrow points to the bipolar cells with their axon terminals located in the proximal portion of the IPL. ONL, outer nuclear layer; INL, inner nuclear layer; IPL, inner plexiform layer; GCL, ganglion cell layer. The scale bar represents 50 µm in **A** (applies to **B**).

For the 5-HTR2a-cre line, in retinal whole mounts, bright tdTomato-expressing cells were observed throughout the retina, especially in the focal plane at the distal portion of the inner nuclear layer (INL; Figure 1A). In the retinal vertical sections, the majority of these cells appeared to be bipolar cells, with the somata located in the distal portion of the INL (Figure 1B). The tdTomato-labeled cell processes were observed through the entire IPL, although the fluorescence signal was more intense in the distal half of the IPL. In addition, sparse axon terminals located in the proximal portion of the IPL were also apparent (marked with arrows in Figure 1B). These terminals resemble the axon terminals of rod bipolar cells with characteristic terminal boutons and location in the proximal margin of the IPL. Furthermore, tdTomato-expressing cells with somata located in the proximal portion of the INL and ganglion cell layer were also observed (Figure 1B). These cells were most likely ganglion cells or displaced amacrine cells [39].

**Figure 2:**
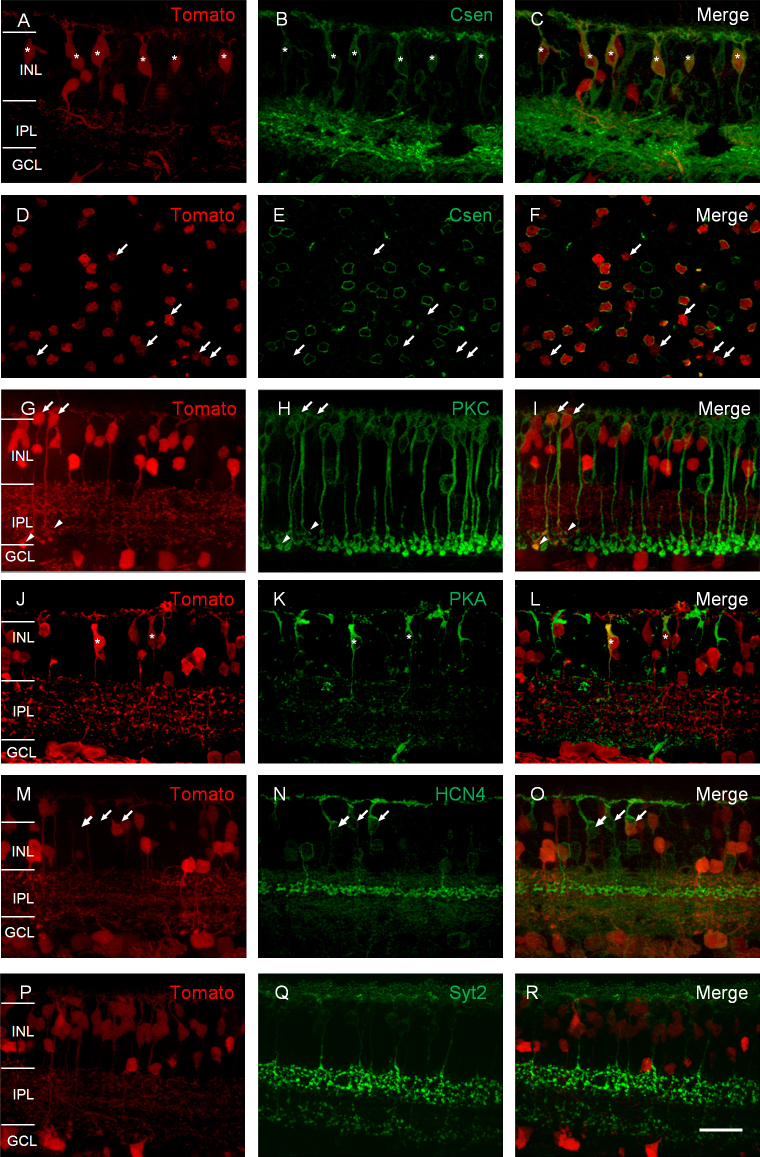
The tdTomato-expressing retinal bipolar cells in the 5-HTR2a-cre mouse line are co-labeled with antibodies specific to type 4 and type 3b cone bipolar cells, and rod bipolar cells. **A**–**C**: In a retinal vertical section, the tdTomato-expressing retina (**A**) was immunostained for calsenilin (**B**). The overlay of A and B is shown in **C**. The double-positive bipolar cells are marked with stars. **D**–**F**: In a retinal whole mount with the focal plane at the distal portion of the inner nuclear layer (INL), the tdTomato-expressing retina (**D**) was immunostained for calsenilin (**E**). The overlay of **D** and **E** is shown in **F**. The majority of the tomato-expressing cells in the distal portion of the INL are calsenilin-positive. The tdTomato-expressing cells that do not show calsenilin staining are marked with arrowheads. The tdTomato-expressing retina was immunostained for PKCα in a retinal vertical section (**G–I**). The double-positive bipolar cells are marked with arrows in the somata and arrowheads pointing to the axon terminals. **J**–**L**: The tdTomato-expressing retina was immunostained for PKARIIβ. Two double-positive cells are marked with stars. The tdTomato-expressing retinal bipolar cells were not labeled by antibodies for HCN4 (**M**–**O**) and Syt2 (**P**–**R**). Scale bars represent 50 µm. ONL, outer nuclear layer; IPL, inner plexiform layer; GCL, ganglion cell layer.

To identify the bipolar cell types in the 5-HTR2a-cre line, we colabeled tdTomato-expressing cells with several bipolar cell type–specific antibodies. Our previous studies showed that the GFP-expressing cells in a 5-HTR2a-GFP transgenic animal line are predominantly type 4 bipolar cells [37]. Therefore, we first labeled the retina with an antibody to calsenilin, a type 4 bipolar cell marker [40]. In the retinal vertical sections, the majority of the tdTomato-expressing cells were immunoreactive for calsenilin (Figure 2A–C; marked with stars at the somata). Labeling with calsenilin in the majority of the tdTomato-expressing bipolar cells was also observed in the retinal whole mounts with the focal plane at the distal portion of the INL (Figure 2D–F); however, a small number of tdTomato-expressing cells were negative for calsenilin staining (marked with arrows). In the retinal whole mounts, approximately 69.5±8.9% (mean±SEM; n=6 retinas) of the tdTomato-expressing cells located in the distal portion of the INL were immunoreactive for calsenilin. Next, we labeled the retina with an antibody against PKCα, a rod bipolar cell marker. In retinal vertical sections, some tdTomato-expressing cells (4.5±2.5%; n=6 retinas) were immunoreactive for PKCα (Figure 2G–I; marked with arrows at the somata and arrowheads at the terminals). Furthermore, we labeled the tdTomato-expressing cells with other bipolar-cell-specific markers. We found that a small number of the tdTomato-expressing cells were immunoreactive for PKAIIβ (Figure 2J–L; marked with stars at the somata), but none were immunoreactive for HCN4 (Figure 2M–O). HCN4 and PKAIIβ antibodies are known to label type 3a and type 3b cone bipolar cells, respectively [41]. In addition, the tdTomato-expressing cells were not labeled with synaptotagmin 2 (Syt2; Figure 2P–R), an antibody that labels type 2 and type 6 cone bipolar cells [10,42].

Taken together, these results indicate that the majority of the tdTomato-expressing bipolar cells in the 5-HTR2a-cre line are type 4 cone bipolar cells. In addition, the tdTomato-expressing cell population includes rod bipolar cells and type 3b cone bipolar cells (see Table 1).

**Table 1:** The summary of bipolar cell types identified in 5-HTR2a-cre and Pcpc2-cre mouse lines.

**Bipolar cell type**								
	1	2/6	3a	3b	4	5	7–9	RB

**Cre line**								
5-HTR2a-cre	-	0	0	few	69.5±8.9%	-	-	4.5±2.5%
Pcpc2-cre	-	Many	0	0	0	-	-	75.5±7.1%

The percentage value for each bipolar cell type was estimated based on the total tdTomato- expressing cells in the distal portion of the inner nuclear layer. Unexamined bipolar cell types are marked with “-.”

### Pcp2-cre mouse line

**Figure 3:**
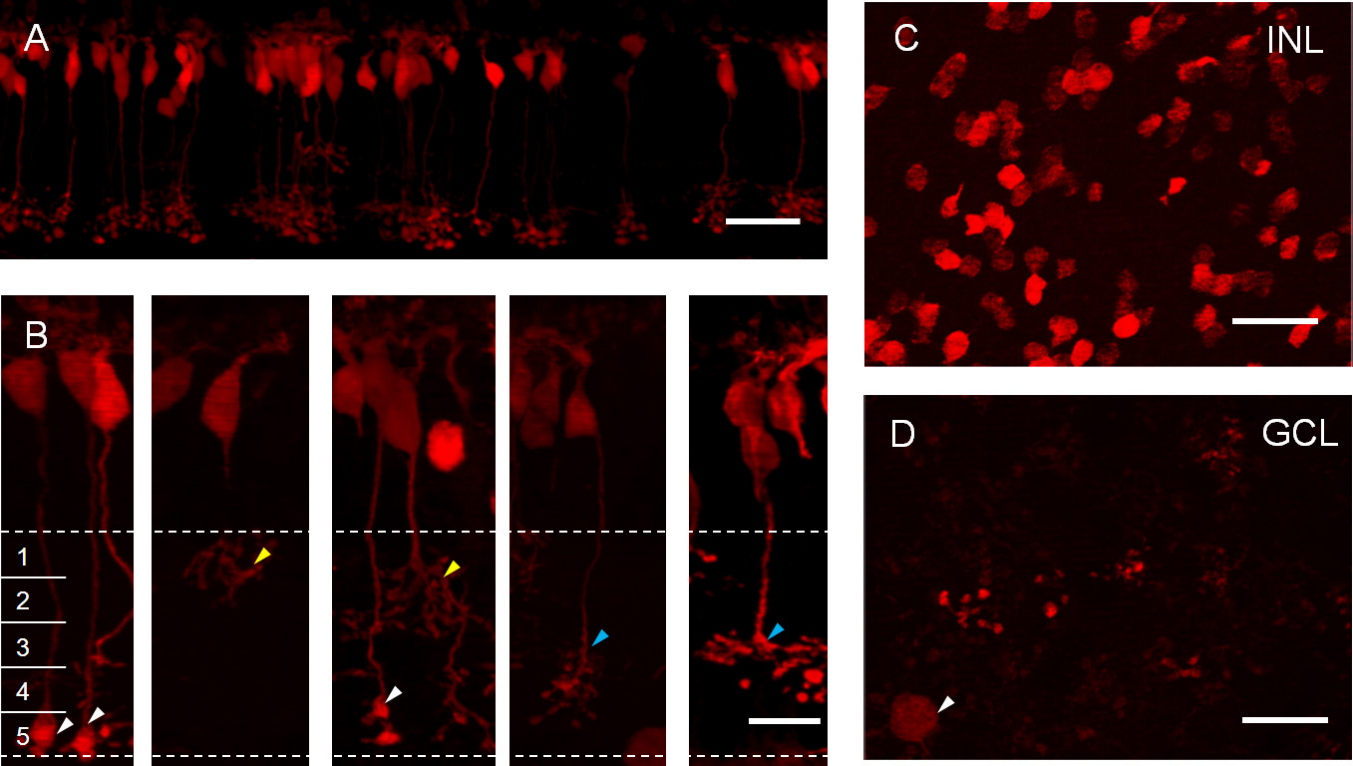
tdTomato-expressing bipolar cells in the Pcp2-cre transgenic mouse line. **B**: Representative bipolar cells in high magnification viewed in retinal vertical sections. The inner plexiform layer (IPL) was divided into five sublaminae. White arrowheads point to the axon terminals of rod bipolar cells. Yellow arrowheads point to the axon terminals of bipolar cells stratified in the distal portion of the IPL. Blue arrowheads point to the axon terminals of bipolar cells stratified in the proximal portion of the IPL. **C**: tdTomato-expressing cells viewed in a retinal whole mount with the focal plane at the INL. **D**: Whole-mount view of the axon terminals of the tdTomato-expressing bipolar cells with the focal plane at the proximal portion of the IPL to the ganglion cell layer. The arrowhead points to a weak tdTomato-expressing ganglion cell. The images in **C** and **D** were taken in the same field. Scale bars represent 25 µm in **A**, **C**, and **D** and 10 µm in **B**. ONL, outer nuclear layer; INL, inner nuclear layer; GCL, ganglion cell layer.

For the Pcp2-cre line, bright tdTomato-expressing cells were also observed throughout the retina. When examined in retinal vertical sections, most of the tdTomato-expressing cells were located in the INL, which were clearly all bipolar cells based on their characteristic morphology (Figure 3A). As shown in Figure 3A, the tdTomato-expressing bipolar cells tend to form clusters. Some of the cells appeared to be rod bipolar cells based on their characteristic terminal buttons located in the proximal margin of the IPL (Figure 3B; marked with white arrowheads). In addition, bipolar cells with axon terminal stratification in the distal portion of the IPL (sublaminae 1 and 2; marked with yellow arrowheads) as well as in the proximal portion of the IPL (sublaminae 4 and 5; marked with blue arrowheads) were also observed, suggesting the presence of labeling in ON and OFF cone bipolar cells. In the retinal whole mount, the tdTomato-expressing bipolar cell somata and axon terminals can also be visualized with the focal plane at the distal portion of the INL (Figure 3C) and the proximal portion of the IPL to the ganglion cell layer (Figure 3D), respectively. In addition, sparsely distributed cells with somata located in the ganglion cell layer were also labeled (marked with an arrowhead in Figure 3D). The characterization of these ganglion cells has been reported [43].

**Figure 4:**
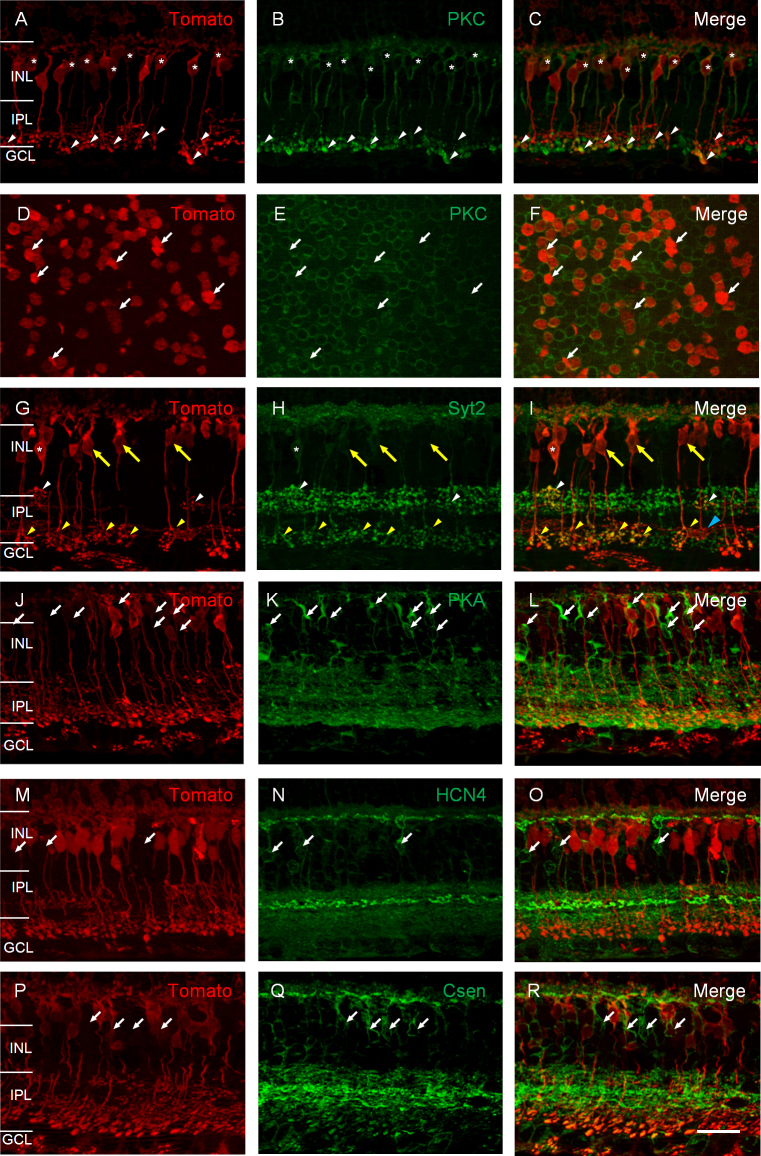
The tdTomato-expressing retinal bipolar cells in the Pcp2-cre mouse line are co-labeled with antibodies specific to rod bipolar cells, type 2 and 6 cone bipolar cells. **A**–**C**: In retinal vertical sections, the tdTomato-expressing retina (**A**) was immunostained for PKCα (**B**). The overlay of **A** and **B** is shown in **C**. The double-positive bipolar cells were marked with stars in the somata and with arrowheads pointing at the axon terminals. **D–F**: In the retinal whole mount with the focal plane in the INL, colabeling with tdTomato and PKCα. PKCα-negative tdTomato-expressing cells are marked with arrows. **G–I**: The tdTomato-expressing retina was immunostained for Syt2. The double-positive bipolar cells with axon terminals stratified at the distal portion of the IPL (type 2 bipolar cells) are marked with white stars in the somata and with white arrowheads pointing at the axon terminals. The double-positive bipolar cells with axon terminals stratified in the proximal portion of the IPL (type 6 bipolar cells) are marked with yellow arrows at the somata and yellow arrowheads at the axon terminals. A tdTomato-expressing bipolar cell with their axon terminals stratified slightly distal to Syt2-positive cells is marked with a blue arrowhead (**I**). The tdTomato-expressing retinal bipolar cells were not found to be labeled by PKARIIβ (**J**–**L**), HCN4 (**M**–**O**), and calsenilin (**P**–**R**). Scale bars represent 50 µm. ONL, outer nuclear layer; INL, inner nuclear layer; IPL, inner plexiform layer; GCL, ganglion cell layer.

To identify the bipolar cell types in the Pcp2-cre line, we colabeled the tdTomato-expressing cells with bipolar cell-specific antibodies. First, many tdTomato-expressing cells were labeled with PKCα and, therefore, are rod bipolar cells as revealed in the retinal vertical sections (Figure 4A–C; marked with stars in the somata and arrowheads at the axon terminals). Colabeling of PKCα and tdTomato was also observed in the retinal whole mounts (Figure 4D–F). Approximately 75.5±7.1% (mean±SEM; n=6) of the tdTomato-expressing bipolar cells were immunoreactive for PKCα, indicating that the majority of the tdTomato-expressing cells were rod bipolar cells, but the presence of PKCα-negative tdTomato-expressing cells is also evident (marked with arrows in Figure 4D–F). These results suggest that other bipolar cell types exist in addition to rod bipolar cells. Indeed, some tdTomato-expressing cells were immunoreactive for Syt2 (Figure 4G–I). Among them, a few of these cells had their axon terminals stratified in the distal portion of the IPL (somata marked with white stars and axon terminals by white arrowheads) and, therefore, were most likely type 2 bipolar cells. The majority of the Syt2-positive cells had axon terminals stratified in the proximal portion of the IPL (somata marked with yellow arrows and axon terminals marked with yellow arrowheads) and thus should be type 6 bipolar cells. The percentage of these two cell types was not determined due to weak labeling of Syt2 at the somata. However, the tdTomato-expressing cells were not labeled by antibodies against PKAIIβ (Figure 4J–L), HCN4 (Figure 4M–O), or calsenilin (Figure 4P–R), indicating that there were no type 3 and type 4 tdTomato-expressing bipolar cells. In addition, there were tdTomato-expressing bipolar cells with axon terminals stratified slightly distal to Syt2-positive type 6 bipolar cells (marked with a blue arrowhead in Figure 4I). These cells might be type 5 and/or 7 cone bipolar cells.

Taken together, these results indicate that the majority of the tdTomato-expressing cells in the Pcp2-cre line are rod bipolar cells. The tdTomato-expressing cells also contain many type 6 cone bipolar cells, some type 2 cone bipolar cells, and possibly type 5 or 7 cone bipolar cells, but not type 3 or 4 cone bipolar cells (see Table 1).

### Chx10-cre mouse line

**Figure 5:**
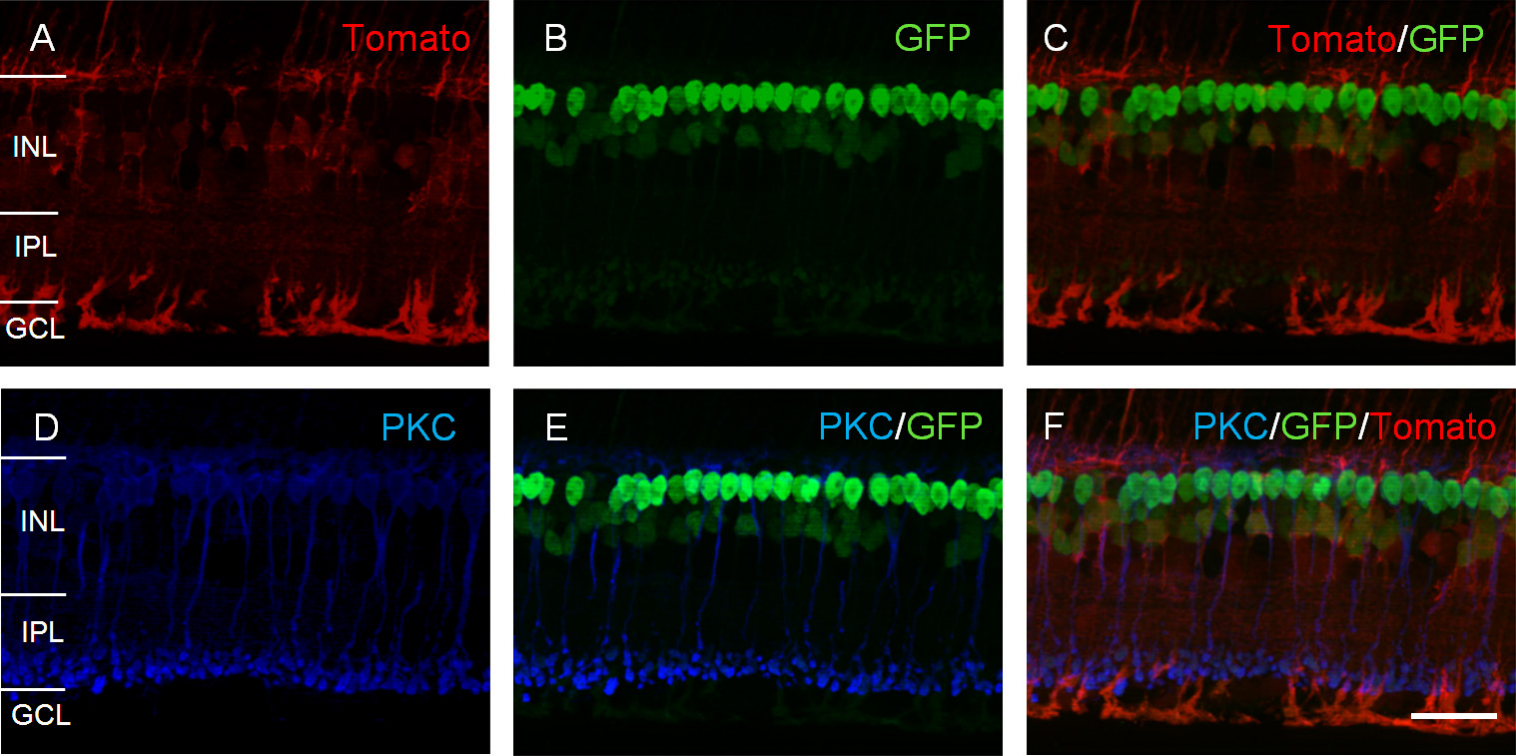
tdTomato-expressing pattern in the Chx10-cre transgenic mouse line. **A–C**: In a retinal vertical section, the expression pattern of tdTomato fluorescence (**A**) is compared with that of GFP-cre. The GFP was enhanced with an antibody to GFP. The overlay of **A** and **B** is shown in **C**. **D–F**: In the same retinal vertical section, the expression pattern of PKC (**D**), which labels rod bipolar cells, is compared with that of GFP and tdTomato. The overlay of PKC and GFP labeling is shown in **E**. The triple overlay of PKC, GFP, and tdTomato labeling is shown in **F**. Scale bars represent 25 µm.

For the Chx10-cre line, bright tdTomato fluorescence was observed throughout the entire retina in the retinal whole-mount preparations (data not shown). In the vertical sections, the tdTomato fluorescence was predominantly located in the Müller cells, based on their characteristic morphology (Figure 5A). Interestingly, this pattern does not fully resemble that of the GFP-cre (enhanced with GFP antibody; Figure 5B,C). In particular, the tdTomato fluorescence was absent in the strongest GFP-labeled cells, with the somata located in the distal portion of the INL and the axon terminals located in the proximal margin of the IPL. These strongest GFP-labeled cells were rod bipolar cells because they showed colabeling with an antibody against PKC (Figure 5D–F). The GFP-labeled cells including weakly labeled Müller cells were visible without antibody enhancement. In addition, previous studies showed that the Cre and GFP immunostaining patterns are the same [32]. The latter would be expected because the transgenic line was generated with a GFP-Cre fusion transgene [29]. Therefore, the bright expression of tdTomato fluorescence in the Müller cells could be explained by a low level of Cre expression in these cells. The lack of tdTomato fluorescence in the strong GFP-Cre-expressing bipolar cells, however, is unexpected. Examination of retinal bipolar cell types in this line, therefore, was not performed further.

### Cre-dependent rAAV-mediated transgene delivery

**Figure 6:**
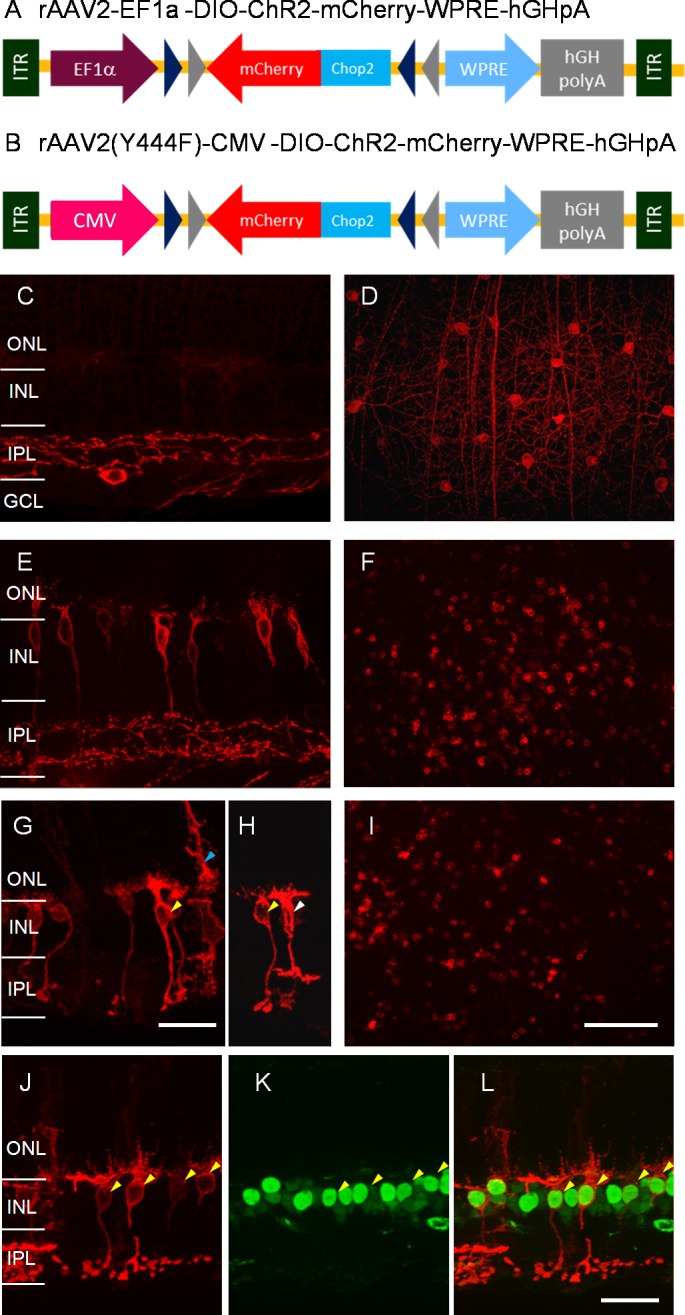
Cre-dependent rAAV-mediated transgene delivery to retinal bipolar cells. **A**–**B**: The Cre-dependent rAAV2 virus vector cassettes that mediate the expression of a double-floxed inverted open-reading frame (DIO) encoding channelrhodopsin-2-mCherry (ChR2-mCherry) with ubiquitous neuronal promoter EF1α (**A**) and with Y444F capsid mutation and a ubiquitous viral promoter, cytomegalovirus (CMV; **B**). WPRE: woodchuck post-transcriptional regulatory element. hGHpA: a human growth hormone polyadenylation sequence. **C**–**D**: The expression of ChR2-mCherry mediated by virus vectors with the vector cassette shown in (**A**) in Pcp2-cre mice, viewed in retinal vertical section (**C**) and in the retinal whole mount with the focal plane at the GCL (**D**). **E**–**F**: The expression of ChR2-mCherry mediated by virus vectors with the vector cassette shown in (**B**) in Pcp2-cre mice, viewed in the retinal vertical section (**E**) and in the retinal whole mount with the focal plane at the INL (**F**). **G**–**I**: The expression of ChR2-mCherry infected by virus vectors with the vector cassette shown in (**B**) in Chx10-cre mice, viewed in the retinal vertical section (**G** and **H**) and in the retinal whole mount with the focal plane at the INL (**I**). **J**–**L**: In the vertical retinal sections, colabeling of GFP (**J**) and mCherry (**K**) of the virus infected retina in Chx10-cre mice. The overlay of **J** and **K** is shown in **L**. The double-positive bipolar cells are marked with arrowheads. Scale bars represent 25 µm in **C**, **E**, **G**, **H**, and **J**–**L** and 100 µm in **D**, **F**, and **I**.

We next evaluated the ability and efficiency of Cre-dependent rAAV-mediated gene delivery for targeting the transgene to Cre-expressing bipolar cells in the retina. We first examined an rAAV2 vector construct carrying a double-floxed inverted open-reading frame (DIO) sequence encoding ChR2-mCherry driven by a ubiquitous neuronal promoter, EFlα (see Figure 6A). The viral vectors were injected intravitreally in the eyes of adult Pcp2-cre mice. This virus vector construct effectively delivered the transgene to retinal ganglion cells but not bipolar cells. As shown in the retinal vertical sections (Figure 6C), bright expression of ChR2-mCherry was observed in a population of Cre-expressing retinal ganglion cells, but expression was barely detected in retinal bipolar cells. The bright expression of ChR2-mCherry in retinal ganglion cells was also evident in the retinal whole mount with the focal plane at the ganglion cell layer (Figure 6D). Because a recent study reported that a capsid mutation of AAV2 (Y444F) can markedly increase the transduction efficacy in distal retinal neurons via intravitreal injection [44], we replaced the virus construct with the capsid mutation. In addition, the EFlα promoter was replaced with a ubiquitous viral promoter, CMV (Figure 6B), because rAAV2 vectors with CMV promoters were observed to show a better transduction efficacy in bipolar cells (data not shown). Indeed, the virus vector with the combination of the Y444F capsid mutation and the CMV promoter resulted in bright expression of ChR2-mCherry in retinal bipolar cells as well as in retinal ganglion cells (Figure 6E). The expression of ChR2-mCherry in retinal bipolar cells was also shown in a retinal whole mount with the focal plane at the INL (Figure 6F).

We also examined the Cre recombination profile of the rAAV2 vectors with the combination of the Y444F capsid mutation and the CMV promoter in the Chx10-cre mouse line because of its unexpected Cre recombination profile using the report mouse line. ChR2-mCherry expression was observed in many bipolar cells, as shown in the retinal vertical sections (Figure 6G,H), and in the retinal whole mount with the focal plane at the INL (Figure 6I). Based on morphological properties and terminal stratification, rod bipolar cells (marked with yellow arrowheads in Figure 6G,H) and cone bipolar cells (marked with a white arrowhead in Figure 6H) were transfected, consistent with Cre expression in the multiple bipolar cell types reported in this line [29]. In addition, ChR2-mCherry expression was also observed in some Müller cells (marked with a blue arrowhead in Figure 6G). Furthermore, we colabeled GFP-cre and mCherry (Figure 6J–L). The results confirm that mCherry indeed targets GFP-cre expressing bipolar cells (marked with arrowheads).

## Discussion

In this study, we examined the Cre/LoxP recombination efficiency and expression patterns of three retinal bipolar cell-expressing Cre transgenic lines by crossing these lines with a strong Cre reporter line. We also examined Cre-dependent rAAV-mediated gene delivery and reported an rAAV vector construct that can deliver the transgene to bipolar cells through intravitreal injection.

Our results show that, for the 5-HTR2a-cre and Pcp2-cre mouse lines, the expression pattern of the transgene gene in bipolar cells mediated by the Cre recombination through the reporter line largely resembles the expression pattern of Cre. However, for both lines, this study revealed additional bipolar cell types are involved in Cre recombination, as discussed below. For the 5-HTR2a-cre mouse line, our results show that the majority of the tdTomato-expressing bipolar cells observed after crossing with the reporter line are type 4 cone bipolar cells, based on the colabeling with antibodies against calsenilin. This result is similar to the previous reported result of a 5-HTR2a-GFP mouse line that was produced using the same BAC promoter construct [37]. In addition, in the 5-HTR2a-cre and 5-HTR2a-GFP lines, a few of the labeled bipolar cells were identified as type 3b cone bipolar cells. Two discrepancies, however, were observed between the tdTomato expression pattern in the 5-HTR2a-cre line and the GFP expression pattern of 5-HTR2a-GFP. First, a small percentage of the tdTomato-expressing cells in the 5-HTR2a-cre line were rod bipolar cells. In addition, many bright tdTomato-expressing third-order neurons were observed in this line. These differences are likely to result from the combination of the highly sensitive Cre recombination and the strong Cre reporter line used in this study. In previous studies of the 5-HTR2a-GFP line, barely visible GFP (after GFP enhancement) was observed in some ON type cone bipolar cells [37]. The current study indicates at least some of these bipolar cells are rod bipolar cells. Additionally, in the 5-HTR2a-GFP line, some weakly labeled cells were observed in the GCL. Therefore, the expression patterns of the transgene in these two lines, Cre and GFP, are likely to be similar, suggesting that the local chromatin environment does not significantly influence the transgene expression profile for the 5-HTR2a BAC promoter.

For the Pcp2-cre line, based on the GFP fluorescence, our previous study showed that the GFP/Cre-expressing bipolar cells were mainly rod bipolar cells [32]. The results of this study, obtained by crossing with the reporter line, also showed that the majority of the tdTomato-expressing bipolar cells are rod bipolar cells, based on the colabeling with an antibody against PKCα. In addition, the current study also revealed the expression of tdTomato in many type 6 cone bipolar cells as well as in some type 2 cone bipolar cells based on colabeling with an antibody against Syt2. Furthermore, our results suggest that there are additional tdTomato labeled ON-type cone bipolar cells. Based on the terminal stratification level, these cells could be type 5 or 7 cone bipolar cells. The observation of these additional tdTomato-expressing bipolar cell types again suggests there is likely weak expression of Cre recombinase in these cells. Although Pcp2 has been known to be rod-bipolar-cell specific, the expression of the transgene in other bipolar cell types in this mouse line is not completely surprising because the Pcp2-cre line was generated by pronuclear injection, in which the transgenic phenotype would be subject to the position effect.

For the Chx10-cre line, the expression pattern of tdTomato resulting from crossing with the Cre reporter mouse was markedly different from that of the GFP/Cre. First, there was strong expression of tdTomato fluorescence in Müller cells although this was not surprising because there is weak expression of GFP/Cre in Müller cells [32] (also see Figure 5). The expression of Cre in a subset of Müller glial cells in the Chx10-cre line was also previously reported [45]. Surprisingly, however, the tdTomato fluorescence was absent in the strongest GFP/Cre-expressing retinal bipolar cells. The transgene expression profile delivered via rAAV vectors was more consistent with the Cre expression in this line [29,32]. We observed robust Cre-mediated transgene expression in multiple types of bipolar cells as well as in some Müller cells in this line through Cre-dependent DIO virus vectors. The reason for the lack of Cre/loxP recombination in the GFP-Cre expressing bipolar cells via the reporter line is not clear. Strong Cre expression might cause rearrangement of genomic DNA or transgene silencing. In addition, the Chx10-cre line is known to display mosaic expression in retinal progenitor cells [29]. The mosaicism might contribute to the unexpected Cre recombination profile observed in this study. Whether this phenomenon is reporter mouse line dependent remains to be determined. Nevertheless, our results point to the importance of the validation of the expression profile through in vivo examination of reporter gene expression [22].

Together, by directly crossing with the Cre reporter mouse line and validating with immunostaining, our studies reveal the existence of multiple Cre-expressing bipolar cell types in two transgenic lines. These two Cre mouse lines could be valuable tools for gene targeting and manipulating bipolar cells in the mouse retina. However, our results suggest that the Chx10-cre mouse line may not be suitable for transgenic mouse line-based gene manipulation but can still be used for virus-based applications.

In this study, we also evaluated the Cre-dependent virus-mediated gene delivery to retinal bipolar cells by intravitreal injection. Our results show that an AAV2 vector construct with the combination of the Y444F capsid mutation and CMV promoter can effectively target the transgene to retinal bipolar cells. Thus, double-floxed inverted AAV2 vectors could provide another valuable tool for gene targeting and manipulating bipolar cells using Cre transgenic mouse lines. There are several advantages in using virus-mediated targeting or delivery. First, using virus-mediated gene delivery allows time-dependent control of gene manipulation. Second, virus-mediated gene delivery can be less subject to the complex problems arising from crossing with reporter lines. As shown in this study, for the Chx10-cre line, the Cre-mediated recombination in retinal bipolar cells could not be achieved by crossing with the reporter mouse line but was achieved through Cre-dependent rAAV vector delivery. Furthermore, combining with promoters and virus serotypes allows differential targeting to different retinal cell populations/types in Cre-transgenic mouse lines.
